# Trends in the incidence of possible severe bacterial infection and case fatality rates in rural communities in Sub-Saharan Africa, South Asia and Latin America, 2010–2013: a multicenter prospective cohort study

**DOI:** 10.1186/s12978-016-0177-1

**Published:** 2016-05-24

**Authors:** Patricia L. Hibberd, Nellie I. Hansen, Marie E. Wang, Shivaprasad S. Goudar, Omrana Pasha, Fabian Esamai, Elwyn Chomba, Ana Garces, Fernando Althabe, Richard J. Derman, Robert L. Goldenberg, Edward A. Liechty, Waldemar A. Carlo, K. Michael Hambidge, Nancy F. Krebs, Pierre Buekens, Elizabeth M. McClure, Marion Koso-Thomas, Archana B. Patel

**Affiliations:** Massachusetts General Hospital for Children, Boston, MA USA; RTI International, Research Triangle Park, North Carolina, USA; Jawaharlal Nehru Medical College, Belgaum, India; Aga Khan University, Karachi, Pakistan; Moi University, Eldoret, Kenya; University of Zambia, Lusaka, Zambia; Institute of Nutrition of Central America and Panama (INCAP), Guatemala City, Guatemala; Institute for Clinical Effectiveness and Health Policy, Buenos Aires, Argentina; Christiana Health Care, Newark, DE USA; Columbia University, New York, NY USA; Indiana University School of Medicine, Indianapolis, IN USA; University of Alabama at Birmingham, Birmingham, AL USA; University of Colorado Health Sciences Center, Denver, CO USA; Tulane School of Public Health and Tropical Medicine, New Orleans, LA USA; Eunice Kennedy Shriver National Institute of Child Health and Human Development, Bethesda, MD USA; Lata Medical Research Foundation, Nagpur, India

**Keywords:** Neonatal sepsis, Low middle income countries, Possible severe bacterial infections, Incidence of neonatal sepsis, Case fatality rates from neonatal sepsis, Global health

## Abstract

**Background:**

Possible severe bacterial infections (pSBI) continue to be a leading cause of global neonatal mortality annually. With the recent publications of simplified antibiotic regimens for treatment of pSBI where referral is not possible, it is important to know how and where to target these regimens, but data on the incidence and outcomes of pSBI are limited.

**Methods:**

We used data prospectively collected at 7 rural community-based sites in 6 low and middle income countries participating in the NICHD Global Network’s Maternal and Newborn Health Registry, between January 1, 2010 and December 31, 2013. Participants included pregnant women and their live born neonates followed for 6 weeks after delivery and assessed for maternal and infant outcomes.

**Results:**

In a cohort of 248,539 infants born alive between 2010 and 2013, 32,088 (13 %) neonates met symptomatic criteria for pSBI. The incidence of pSBI during the first 6 weeks of life varied 10 fold from 3 % (Zambia) to 36 % (Pakistan), and overall case fatality rates varied 8 fold from 5 % (Kenya) to 42 % (Zambia). Significant variations in incidence of pSBI during the study period, with proportions decreasing in 3 sites (Argentina, Kenya and Nagpur, India), remaining stable in 3 sites (Zambia, Guatemala, Belgaum, India) and increasing in 1 site (Pakistan), cannot be explained solely by changing rates of facility deliveries. Case fatality rates did not vary over time.

**Conclusions:**

In a prospective population based registry with trained data collectors, there were wide variations in the incidence and case fatality of pSBI in rural communities and in trends over time. Regardless of these variations, the burden of pSBI is still high and strategies to implement timely diagnosis and treatment are still urgently needed to reduce neonatal mortality.

**Trial registration:**

The study was registered at ClinicalTrials.gov (NCT01073475).

## Background

The annual global burden of mortality in children under age 5 years decreased by 36 % from 9.9 million in 2000 to 6.3 million in 2013, mostly due to reductions in mortality due to pneumonia, diarrhea and measles in children aged 2–59 months [1]. Over the same time period, there was only a 30 % reduction in the annual neonatal mortality rate from 4 million to 2.8 million, so that the proportion of under age 5 deaths that occurred in neonates increased from 38 to 44 % [2]. Most of the neonatal deaths continue to be attributed to preterm birth, intrapartum related complications (birth asphyxia) and neonatal sepsis/meningitis/pneumonia due to possible severe bacterial infections (pSBI) [1]. The recently published studies of simplified antibiotic regimens for treatment of neonatal pSBI provide a potential way to reduce part of the burden of neonatal mortality in settings where hospitalization is not accepted or available [3,4], but the impact of these new regimens and optimal ways to make them available globally is not clear, [5,6] because there is limited information on the incidence and case fatality rates of pSBI. To our knowledge, only one systematic review and meta-analysis estimates the global incidence and mortality due to neonatal infection, in one year - 2012 [7]. There is an important need to confirm these estimates of neonatal pSBI incidence and case fatality rates using prospectively collected, population based data, in which the outcomes of all pregnancies are recorded and to evaluate recent trends over time, particularly as facility births and survival of preterm infants increase.

The diagnosis of possible severe bacterial infection/neonatal sepsis is challenging even in well-equipped tertiary care facilities in resource rich settings [8,9]. In resource limited settings where there is a lack of access to microbiologic, hematologic and biochemical laboratory studies, the World Health Organisation’s (WHO) Integrated Management of Childhood Illness (IMCI) algorithm is used to make a clinical diagnosis of possible severe bacterial infection (pSBI), which includes sepsis, meningitis and pneumonia in young infants and neonates [10]. However, the signs can be difficult to detect and non-specific. The algorithm, initially developed in the first WHO Young Infants Study in the 1990s, found 14 clinical signs and symptoms that predicted isolation of bacteria in blood or cerebrospinal fluid, or culture positive severe bacterial disease [11]. These signs and symptoms were simplified in the second WHO Young Infants Clinical Signs Study (YICSS) published in 2008 [10]. The YICSS evaluated clinical signs in neonates presenting to primary health care facilities, as detected by primary care health workers, in 6 countries (Bangladesh, Bolivia, Ghana, India, Pakistan, and South Africa). Presence of any one of seven clinical signs and symptoms predicted severe illness (based on an expert pediatrician’s assessment) and was associated with a sensitivity and specificity of 85 and 75 % respectively in 0–6 day old neonates and 74 and 79 % respectively in infants aged 7–59 days [10]. Since the YICSS signs and symptoms were not evaluated against blood or cerebrospinal fluid culture results, the diagnosis likely includes respiratory distress associated with preterm birth, birth asphyxia, and viral respiratory infections, but based on available data, neonates with these signs and symptoms should be treated for pSBI.

The Eunice Kennedy Shriver National Institute of Child Health and Human Development (NICHD) Global Network (GN) for Women and Children’s Health Research supports a population based Maternal and Newborn Health (MNH) Registry of pregnant women and their babies living in rural communities in six low and lower middle income countries. The Registry has focused on documentation of pregnancy histories, details of labor and delivery, accurate and timely measurement of birth weight and early and late neonatal morbidity and mortality through day 42 of life, including signs and symptoms similar to those used in the YICSS, and cause of death [12]. We used data from live born neonates collected by the MNH Registry between 2010 and 2013 to estimate the incidence of pSBI using the clinical signs and symptoms from YICSS, the case fatality rate, and trends in pSBI incidence and mortality over time.

## Methods

### Ethics statement

The appropriate Institutional Review Boards and Ethics Research Committees of all participating institutions and the Ministries of Health of the respective countries reviewed and approved participation in the MNH Registry. Prior to initiation of the Registry, approval was also sought from the participating communities through sensitization meetings. Study participants were enrolled in the Registry after they provided written informed consent. No monetary reimbursements were provided to either individual participants or to communities. A Data Monitoring Committee, appointed by the NICHD, oversaw and reviewed the Registry at annual meetings. The MNH Registry study was registered at ClinicalTrials.gov (NCT01073475).

### Study design, setting and participants

The MNH Registry is an ongoing prospective multicenter cohort study of pregnant women and their babies who were residents or resided for at least 4 weeks in rural communities (clusters) in Argentina, Guatemala, 2 states in India, Kenya, Pakistan and Zambia [12]. Details of the study sites are provided in Table 1 [13]. Pregnant women are recruited as early as possible during pregnancy and followed through day 42 postpartum to obtain details about the pregnancy, labor and delivery and the health of the mother and infant. Data are collected by trained staff at enrolment and again at within 7 days of delivery and at 42 days after delivery and reviewed by medical officers. On enrolment, date of last menstrual period, estimated delivery date, age, education, parity, and status of last child are collected. Information collected within 7 days of delivery includes prenatal care, complications occurring during pregnancy, mode and location of delivery, status of the mother and newborn following delivery, sex and birth weight of the newborn, and neonatal conditions. Maternal and newborn status and neonatal adverse conditions between delivery and 42 days after birth are collected at the second postpartum visit. Additional study details have been described previously [12].

**Table 1:** Details of the study sites in the MNH registry

Location	Type	Community Setting	Human Development Index ^a^
South Asia			
India (Belgaum) – South West Karnataka	Semi-urban	The study area includes 24 primary health centers (clusters). Each is managed by a physician medical officer who works with nursing staff and auxiliary nurse midwives in associated sub-centers, the most peripheral outpost of the health care services. There are 3 tertiary care hospitals and eight secondary care hospitals serving the region as referral hospitals staffed by obstetricians, pediatricians and nurses. In addition to these public sector health facilities, there are several private sector maternity facilities within the site catchment area.	0.508
India (Nagpur) – Eastern Maharashtra	Semi-urban and rural	The study area includes 20 primary health centers, each served by physician medical officers and nurses. These areas include 119 sub-centers where basic maternal and child care are provided. Referral care is provided in ten tertiary hospitals (2 public sector and 8 private sector), and 129 secondary hospitals (27 public sector hospitals and 102 private nursing homes).	0.549
Pakistan – two of five sub districts in Thatta, Sindh province	Semi-urban and rural	The study area includes over 75 health facilities, both public sector and private fee-for-service, providing maternal and child health services. This includes 47 primary health clinics in 24 clusters, 25 secondary care facilities and 3 referral hospitals. Care in health clinics is typically provided by either paramedical staff, including nurses and lady health visitors, or non-specialist physicians. Obstetricians provide care in referral hospitals.	0.595
Sub-Saharan Africa			
Kenya – western Kenya in Busia, Bungoma and Kakamega counties	Rural	The study area is served by 23 health facilities in 16 clusters, most operated by the government and staffed by nurse-midwives and clinical officers and a single medical officer. Three hospitals function as referral hospitals. Most physicians are generalists, with a small number of trained obstetricians and pediatricians.	0.570
Zambia – Kafue and Chongwe districts, East Zambia	Rural	There are ten clusters, eight of which have health posts. Care is provided primarily by nurse midwives in the health posts and by traditional birth attendants for home births. There are 3 district hospitals and a referral hospital in Lusaka. Specialty physicians are available in the referral hospital only.	0.465
Latin American Sites			
Argentina – Corrientes and Santiago del Estero Provinces, North Argentina	Semi-urban and rural	There are six clusters, three in each province. Each cluster corresponds to a department (municipality) in which the vast majority of the births occur at a publicly-funded secondary level hospital. Care is provided primarily by physicians and midwives. There are 2 Provincial referral hospitals located in provincial capital cities.	0.828
Guatemala – Chimaltenago region, Western Highlands	Rural	There are 18 clusters served by one referral hospital, 30 health centers, and 42 health posts. Maternal and infant care in the hospital is provided mainly by obstetricians and general physicians, in health centers by physicians and nurses, and in health posts by auxiliary nurses	0.679

^a^ The human development index is a composite statistic of life expectancy, education, and income per capita indicators that are used to rank countries into four tiers of human development

For this secondary data analysis, we included infants born alive between January 1, 2010 and December 31, 2013 to the pregnant women enrolled in the seven sites participating in the MNH Registry. We excluded infants with missing birth weights, as well as those with birth weight measured more than 7 days after delivery or with unknown timing of measurement. We excluded babies with missing birth weights as these were rare in the MNH registry (<1 %) due to a focus on collection of birth weight data. The data collection forms changed at the end of 2013 with symptoms used to define pSBI no longer collected. Infants for whom the 42 day assessment was conducted using the revised forms were excluded from analysis. During the study period, women were enrolled in a total of 118 communities (clusters) in the seven sites: Argentina, 6 clusters; Guatemala, 18; Kenya, 16; Zambia, 10; Belgaum, India, 24; Nagpur, India, 20; Pakistan, 24. In Argentina, Kenya, Zambia, and Nagpur all clusters participated throughout the 4 year study period. However, in Guatemala, Belgaum, India and Pakistan, only 9 of 18 clusters, 16 of 24 and 20 of 24 clusters respectively participated throughout the 4 year study period.

### Study outcomes

#### Possible severe bacterial infection (pSBI)

Estimates of pSBI during the first 6 weeks of life were derived retrospectively for all live born infants using symptoms reported on the data collection forms based on the WHO YICSS criteria [10] to the extent possible given the information recorded in the registry. The presence or absence of each of the following symptoms after delivery was recorded in the registry, with a “yes” response considered consistent with pSBI: breathing problems/difficulty breathing, feeding problems/stopped suckling/feeding, high fever (>38 ° C or estimated by touch), hypothermia (<35 ° C or estimated by touch), convulsions, and bleeding/pus-like discharge from umbilicus (Table 2). The MNH Registry variables were not designed for the diagnosis of pSBI specifically, but to standardize the data collection on key outcome variables in pregnant women, mothers and neonates across the Global Network sites. There was an initial standardized training conducted with the Site Principal Investigators (PIs) in the US and then site specific trainings and re-trainings conducted by the Site PIs to ensure consistency of the information collected across sites and over time. In addition, RTI (Data Coordinating Center) monitored MNH variables over time for deviations and unusual patterns and when these occurred, they were brought to the attention of the site PI. In each location the site PI was assisted by a medically trained registry administrator who worked with the local data collectors (mostly medical officers) to ensure that data were collected consistently within sites and over time. The registry administrator and site PI conducted re-trainings at least once a year and more frequently if deviations or unusual patterns in data collection were observed.

**Table 2:** Signs and symptoms of possible severe bacterial infection in the MNH registry

WHO YICSS Signs and Symptoms	GN MNH Registry Signs and Symptoms
History of difficulty feeding	Feeding problems; Stopped suckling/feeding
History of convulsions	Convulsions
Movement only when stimulated	Not collected
Respiratory rate of 60 breaths per minute or more	Breathing problems; Difficulty breathing
Severe chest indrawing	Breathing problems; Difficulty breathing
Temperature ≥ 37.5 ° C	High fever (>38 ° C)
Temperature < 35.5 ° C	Hypothermia (< 35 ° C)
	Other • Bleeding/pus-like discharge from umbilicus • Infection recorded as cause of death • Text indicating infection, sepsis, possible sepsis, septic conditions, meningitis, pneumonia

The specific signs and symptoms of pSBI are challenging, even in resource intense studies [3,4,10] because they are based in part on parent report (e.g., history of difficulty breathing and convulsions). The MNH registry did not collect data on movement when stimulated. The YICSS measured respiratory rates and trained study staff on detection of severe chest indrawing, while the MNH registry reported that any type of breathing problem was either present or absent. Temperature was measured in the YICSS but in some Global Network sites was not actually measured and was only estimated by either the mother or medical officer. To improve the accuracy of the diagnosis of pSBI in the MNH registry, infants who died with cause of death coded “infection” were also counted as having pSBI. Additionally, text fields used to record information about symptoms and diagnoses as well as cause of death were reviewed. Infants with any of the symptoms listed above, or the words “infection”, “sepsis”, “possible sepsis”, “septic” (e.g., a septic condition such as septic rash, septic umbilicus), “meningitis”, and/or “pneumonia” in any of the text fields were counted as having pSBI. All information in the MNH registry was collected by the medical officers who may or may not have seen the child when they had the specific symptom consistent with pSBI, but the medical officer was responsible for determining whether the MNH definition of the sign or symptom was met based on questioning of the mother or data collector, as required by the MNH training. At most study locations there were no charts that could be reviewed for additional information. The number of pSBI episodes and the exact timing of infection could not be determined, as dates of diagnosis were not recorded. Microbiologic confirmation of pSBI was not possible in these rural communities and facilities.

#### Case fatality

Neonatal/early infant case fatality after diagnosis of pSBI was defined as the number of deaths due to any reported cause among infants with pSBI occurring up to 6 weeks after birth divided by the number of infants with pSBI. The cause of death was determined by the registry administrator who conducted a basic death audit, per the MNH manual of operations, by interviewing the birth attendant, and/or family members and the mother, whenever possible.

#### Covariates

Covariates examined included maternal age, maternal education, location of delivery, mode of delivery, infant birth weight, infant sex, and early initiation of breast feeding within an hour of delivery. A hospital was defined as a residential establishment equipped with inpatient facilities for 24-h medical and nursing care and staffed with at least one physician providing essential obstetrical/neonatal care (for example, the ability to provide a caesarean section). A clinic/health center was defined as a facility that provided (ambulatory) medical services to a specific group in a population and that did not have caesarean section capability. Births that occurred at home, whether at the mother’s home, a birth attendant’s home, or a relative’s home, were grouped together for analysis. Other delivery locations were infrequent. Birth weight was recorded using locally available scales, calibrated per local facility protocols.

### Statistical analysis

Maternal, delivery, and neonatal characteristics were compared descriptively across the study sites and over the years studied at each site. The cumulative incidence of pSBI in the first 6 weeks of life was defined as the proportion of infants who developed pSBI during the 42 day follow-up period divided by the number of infants studied, with 95 % confidence intervals estimated using the normal approximation to the binomial distribution. Crude estimates of pSBI incidence and case fatality rates are reported in text and figures. Percentages of infants with each symptom of pSBI were also compared descriptively between the sites.

Statistical testing was conducted using logistic regression models to compare incidence of pSBI across study sites, and to examine trends in the proportion with pSBI by birth year and trends in the proportion with pSBI who died at each study site, while adjusting for covariates. Adjusted analyses were utilized to compare incidence and assess trends after accounting for differences across site and over time in the distribution of maternal, delivery, and neonatal characteristics potentially impacting risk of pSBI. All models included effects for study site, maternal age (<20, ≥ 20), maternal education (no formal, primary, secondary/university), cesarean section delivery (yes, no), facility birth (hospital/clinic/health center, home/other), infant sex (male, female), infant birth weight (<2500, ≥2500 g), and initiation of breastfeeding within 1 h of delivery (yes, no). A test of the null hypothesis that the incidence of pSBI was the same at all sites against the alternative that the incidence of pSBI differed across the sites was conducted using a main effects model with statistical significance determined by the Score chi-square test for study site. Site-specific tests of the null hypothesis that pSBI incidence remained stable during the study period against the alternative that incidence changed, and similar tests of trend in case fatality rates, were conducted using data from all sites in models that included the main effects above plus birth year and all 2 way interactions between study site and each covariate. Birth year was treated as a continuous variable in order to assess linear trend. Contrasts involving the site by birth year interaction were used to assess whether or not changes over time were significant at each site with statistical significance determined by Wald chi-square tests. Analyses examining trends in pSBI incidence by birth year and trends in the case fatality rate were repeated excluding clusters in Guatemala, Belgaum, and Pakistan that did not participate all four years. In a secondary analysis, interactions between site, birth year and facility birth were added to the primary pSBI trends model in order to test whether trends were the same or differed by location of delivery at each site. Generalized estimating equations were used in all models to produce robust standard errors that accounted for clustering of women and infants in geographic areas (“clusters”) at each study site with an independent working correlation structure specified. Just under 2 % of the data were excluded from models due to missing values of covariates. Variables were assumed to be missing at random. Analyses were implemented using SAS version 9.3 (SAS Institute, Inc., Cary NC).

## Results

### Study population

**Fig. 1:**
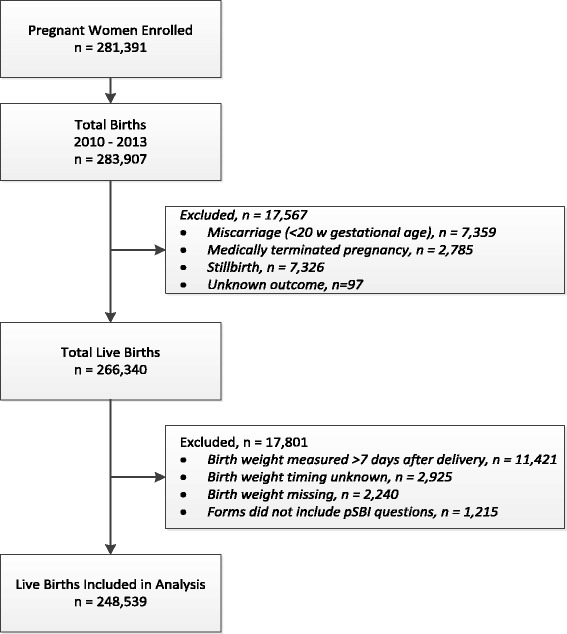
Study flow chart: neonates and young infants included in the maternal and newborn health registry and assessed for possible severe bacterial infection

The GN MNH Registry included 266,340 live births between 2010 and 2013. After exclusions, a total of 248,539 live born infants with birth weight measured within 7 days after delivery were included in the analysis (Fig. 1). By 6 weeks after delivery, 241,623 infants were still alive (97.2 %), 5679 (2.3 %) had died, and 6 week status was unavailable for 1,237 (0.5 %). The percent of infants who died ranged from 1.2 % in Argentina to 3.0 % in Pakistan (Table 3). Median age at death for those who died was 2 to 5 days old depending on study site.

**Table 3:** Six-week vital status for live born infants 2010–2013 by site

	Argentina	Guatemala	Kenya	Zambia	Belgaum, India	Nagpur, India	Pakistan
Number of live births	7,888	26,022	33,724	26,272	77,440	38,303	38,890
Six-week vital status, *n* (%)							
Survived	7,744 (98.2)	25,184 (96.8)	33,239 (98.6)	25,571 (97.3)	75,515 (97.5)	37,218 (97.2)	37,152 (95.5)
Died	96 (1.2)	662 (2.5)	427 (1.3)	463 (1.8)	1,922 (2.5)	935 (2.4)	1,174 (3.0)
Missing	48 (0.6)	176 (0.7)	58 (0.2)	238 (0.9)	3 (0.0)	150 (0.4)	564 (1.5)
Age at death for infants who died							
Median day (p25, p75)	3 (1, 8)	5 (2, 16)	3 (1, 12)	2 (1, 7)	3 (1, 7)	3 (1, 8)	5 (2, 15)

### Maternal, delivery and neonatal characteristics at each study site

The average age of mothers enrolled ranged from 22 years in Belgaum to 27 years in Pakistan (Table 4). In Argentina, Kenya, and Nagpur only 3 % of mothers had no formal schooling. Ten percent of mothers in Zambia had no formal schooling, 19 % in Guatemala, and 21 % in Belgaum, while in Pakistan 83 % of mothers had no formal schooling (Table 4). A secondary or university level education was achieved by 79 % in Nagpur, 46 % in Belgaum, 35 % in Zambia, 34 % in Argentina, 26 % in Kenya, 19 % in Guatemala, and only 9 % in Pakistan. The majority of mothers had at least one antenatal care visit with the lowest percent in Pakistan (85 %) and nearly 100 % in Zambia, Belgaum, and Nagpur.

**Table 4:** Maternal, delivery and neonatal characteristics of live born infants 2010–2013 by site

Characteristic, *n* (%)	Argentina	Guatemala	Kenya	Zambia	Belgaum, India	Nagpur, India	Pakistan
Number of live births	7,888	26,022	33,724	26,272	77,440	38,303	38,890
MATERNAL							
Maternal age, y							
Mean (SD)	24.5 (6.6)	26.1 (6.6)	24.2 (5.6)	24.7 (6.6)	22.7 (3.0)	23.5 (2.9)	27.6 (4.8)
< 20	2,156 (27)	4,299 (17)	7,308 (22)	6,633 (25)	7,377 (10)	750 (2)	1,446 (4)
20–35	5,114 (65)	19,034 (73)	24,970 (74)	17,528 (67)	69,860 (90)	37,419 (98)	35,150 (91)
> 35	587 (7)	2,675 (10)	1,391 (4)	2,060 (8)	129 (< 1)	106 (< 1)	2,149 (6)
Maternal education - highest level							
No formal schooling	201 (3)	4,828 (19)	1,013 (3)	2,691 (10)	15,758 (21)	1,169 (3)	32,284 (83)
Primary	4,893 (63)	16,343 (63)	23,937 (71)	14,328 (55)	25,497 (33)	6,604 (17)	2,917 (8)
Secondary	2,617 (33)	4,563 (18)	7,479 (22)	8,608 (33)	28,432 (37)	22,752 (59)	2,234 (6)
University	117 (1)	277 (1)	1,248 (4)	468 (2)	7,154 (9)	7,731 (20)	1,287 (3)
One or more antenatal visit							
Yes	7,445 (95)	25,582 (98)	32,838 (97)	26,139 (100)	77,186 (100)	38,259 (100)	32,842 (85)
No	357 (5)	411 (2)	867 (3)	111 (< 1)	56 (< 1)	16 (< 1)	5,908 (15)
DELIVERY							
Mode of delivery							
Vaginal	5,072 (64)	20,596 (79)	33,195 (98)	25,976 (99)	66,242 (86)	30,625 (80)	35,584 (92)
Cesarean section	2,813 (36)	5,425 (21)	529 (2)	296 (1)	11,198 (14)	7,678 (20)	3,304 (8)
Location of delivery							
Hospital	7,805 (99)	11,534 (44)	4,524 (13)	3,368 (13)	52,519 (68)	26,131 (68)	10,679 (27)
Clinic/health center	18 (< 1)	1,307 (5)	10,054 (30)	13,131 (50)	20,352 (26)	10,699 (28)	9,932 (26)
Home	36 (< 1)	13,131 (50)	18,885 (56)	9,501 (36)	4,234 (5)	1,454 (4)	18,075 (46)
Other	24 (< 1)	50 (< 1)	260 (1)	272 (1)	297 (< 1)	17 (< 1)	187 (< 1)
Clean razor used to cut cord							
Yes	7,845 (100)	16,171 (68)	33,587 (100)	25,791 (99)	75,907 (100)	38,047 (100)	38,451 (99)
No	24 (< 1)	7,522 (32)	130 (< 1)	281 (1)	274 (< 1)	108 (< 1)	393 (1)
Birth attendant used new gloves							
Yes	7,840 (100)	25,457 (99)	33,189 (98)	25,814 (99)	75,140 (97)	37,383 (99)	30,312 (78)
No	33 (< 1)	303 (1)	528 (2)	246 (1)	1,955 (3)	472 (1)	8,530 (22)
NEONATAL							
Multiple birth							
Yes	104 (1)	347 (1)	769 (2)	430 (2)	1,067 (1)	565 (1)	796 (2)
No	7,767 (99)	25,675 (99)	32,953 (98)	25,842 (98)	76,367 (99)	37,728 (99)	38,065 (98)
Infant sex							
Male	4,071 (52)	13,254 (51)	17,092 (51)	13,830 (53)	40,211 (52)	19,966 (52)	20,369 (52)
Female	3,810 (48)	12,764 (49)	16,632 (49)	12,431 (47)	37,227 (48)	18,333 (48)	18,509 (48)
Birth weight, g							
< 1500	64 (1)	155 (1)	61 (< 1)	96 (< 1)	612 (1)	355 (1)	257 (1)
1500–2499	399 (5)	3,228 (12)	892 (3)	1,212 (5)	9,806 (13)	5,439 (14)	5,579 (14)
2500–2999	1,384 (18)	10,151 (39)	6,607 (20)	6,854 (26)	38,785 (50)	22,551 (59)	15,019 (39)
3000–3499	3,121 (40)	9,375 (36)	15,605 (46)	12,104 (46)	23,434 (30)	8,525 (22)	13,649 (35)
3500+	2,920 (37)	3,113 (12)	10,559 (31)	6,006 (23)	4,803 (6)	1,433 (4)	4,386 (11)
Infant breastfed within 1 h of delivery							
Yes	7,054 (90)	19,319 (75)	27,867 (83)	24,037 (92)	63,425 (85)	32,786 (86)	9,492 (24)
No	770 (10)	6,608 (25)	5,818 (17)	2,108 (8)	10,987 (15)	5,344 (14)	29,357 (76)

Caesarean section delivery was infrequent at the African sites (1–2 %) and most frequent in Argentina (36 %). Nearly all women in the Argentina site (99 %) delivered in hospital. Over 90 % of births in the Indian sites occurred in a facility, compared to 63 % in Zambia, 53 % in Pakistan and less than half of the births in Guatemala and Kenya. A clean razor was used to cut the baby’s cord and new gloves were used by the birth attendant in almost all deliveries, except in Guatemala, where use of a clean razor was reported for 68 % of deliveries (99 % by 2013) and in Pakistan where use of new gloves was reported for 78 % of births.

Only 1–2 % of infants at each site were from a multiple birth, and slightly over half were male. Mean birth weight ranged from 2,677 g in Nagpur to 3,303 g in Argentina. In Guatemala 75 % of infants were breastfed within 1 h of delivery, 83 % in Kenya, 85–86 % in the Indian sites, and 90 % or more in Argentina and Zambia, while in Pakistan only 24 % were breastfed within an hour of birth.

### Changes in maternal, delivery and neonatal characteristics by birth year at each site

In general, maternal educational level increased during the period 2010–2013 at all sites. The proportion of facility births remained at 99 % in Argentina throughout the period. At the other sites the proportion of facility births increased: from 39 % in 2010 to 58 % in 2013 in Guatemala, 38 to 52 % in Kenya, 55 to 71 % in Zambia, 92 to 96 % in Belgaum, 90 to 99 % in Nagpur, and 49 to 62 % in Pakistan. The percent of cesarean section deliveries increased at all sites except Zambia where only 1 % of infants were delivered by cesarean section, from 27 to 40 % of infants in Argentina, 16 to 26 % in Guatemala, 1 to 2 % in Kenya, 10 to 19 % in Belgaum, 18 to 23 % in Nagpur, and 7 to 12 % in Pakistan. The percent of infants who were breastfed within 1 h of delivery remained about 90 % in Argentina and 83 % in Kenya throughout the 4 year period, increased from 85 % in 2010 to 93 % in 2013 in Zambia, and decreased from 80 to 67 % in Guatemala, from 90 to 77 % in Belgaum, 92 to 84 % in Nagpur, and from 31 to 15 % in Pakistan.

### pSBI incidence by site

**Fig. 2:**
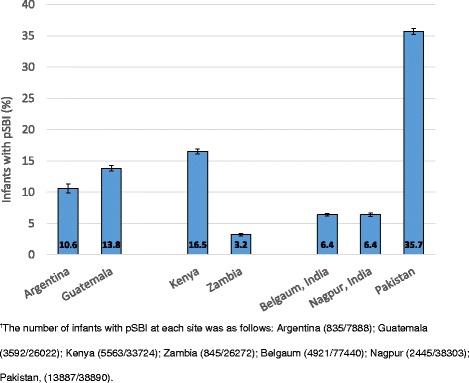
Cumulative incidence of possible severe bacterial infection (pSBI) through 6 weeks of life in live born infants by study site

During the study period, 32,088 of the 248,539 live born neonates met our criteria for pSBI during the first 6 weeks of life (12.9 %, 95 % CI 12.8–13.0 %). The percent of infants with pSBI varied widely by study site, *p* < 0.001, from a low of 3.2 % (95 % CI 3.0–3.4 %) in Zambia to 6.4 % (95 % CI 6.2–6.5 %) in Belgaum, 6.4 % (95 % CI 6.1–6.6 %) in Nagpur, 10.6 % (95 % CI 9.9–11.3 %) in Argentina, 13.8 % (95 % CI 13.4–14.2 %) in Guatemala, 16.5 % (95 % CI 16.1–16.9 %) in Kenya, and a high of 35.7 % (95 % CI 35.2–36.2 %) in Pakistan (Fig. 2).

**Fig. 3:**
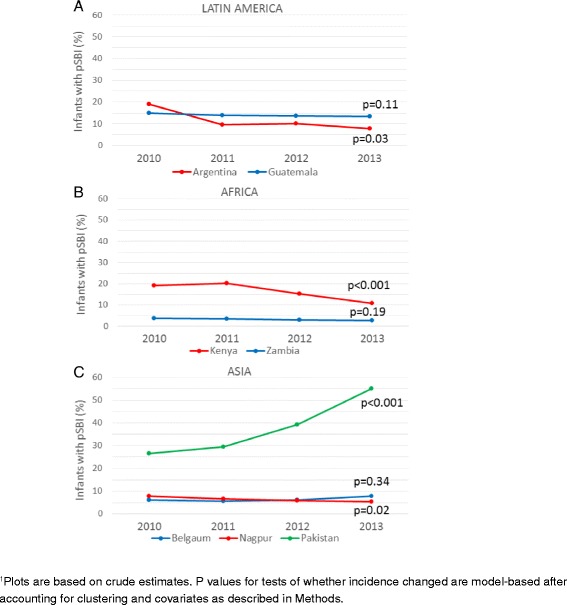
Trends in cumulative incidence of possible severe bacterial infection through 6 weeks of life by study site, 2010–2013

Incidence of pSBI in the first 6 weeks changed significantly over time at some sites (Fig. 3). The proportion of infants with pSBI decreased in Argentina, where nearly all births occurred in hospital, from 19.0 % (95 % CI 16.4–21.6 %) in 2010 to 7.9 % (95 % CI 6.2–9.7 %) in 2013 (adjusted *p* = 0.03 accounting for clustering and maternal and neonatal covariates). In Kenya incidence of pSBI decreased from 19.1 % (95 % CI 18.2–19.9 %) in 2010 to 10.7 % (95 % CI 10.1–11.4 %) in 2013 (adjusted *p* < 0.001), with decreases occurring among infants born at home as well as in a facility [facility births: 21.4 % (20.0–22.9 %) to 11.9 % (10.9–12.9 %); home births: 17.6 % (16.5–18.6 %) to 9.5 % (8.5–10.4 %); adjusted *p* = 0.91 for a difference in trend]. In Nagpur, India, the proportion with pSBI decreased for infants born in a facility [7.8 % (95 % CI 7.2–8.4 %) in 2010 to 5.3 % (95 % CI 4.9–5.8 %) in 2013, adjusted *p* = 0.01] but not for those born at home [6.6 % (5.2–8.5 %) to 17.4 % (10.4–27.5 %); adjusted *p* = 0.35]. In Guatemala there was a small non-significant decrease in the proportion of infants with pSBI [14.9 % (13.7–16.0 %) in 2010 to 13.5 % (12.8–14.1 %) in 2013, adjusted *p* = 0.11]. However, after exclusion of clusters not participating all 4 years the change in Guatemala became statistically significant [14.1 % (12.9–15.4 %) in 2010 to 12.0 % (11.2–12.8 %) in 2013, adjusted *p* = 0.03] and these changes did not differ significantly for home or facility births [facility births: 16.6 % (14.4–18.7 %) to 15.0 % (13.8–16.3 %); home births: 12.7 % (11.3–14.2 %) to 9.1 % (8.1–10.1 %); adjusted *p* = 0.27 for a difference in trend). There was no significant change in the overall incidence of pSBI in the first 6 weeks of life during the study period in Zambia [3.8 % (3.3–4.2 %) in 2010 to 2.8 % (2.3–3.2 %) in 2013, adjusted *p* = 0.19) or in Belgaum [6.2 % (5.9–6.5 %) in 2010 to 7.7 % (7.3–8.1 %) in 2013, adjusted *p* = 0.34; with exclusion of clusters not participating all 4 years, adjusted *p* = 0.35]. In contrast, in Pakistan the proportion of neonates/young infants with pSBI increased from 26.7 % (95 % CI 25.9–27.5 %) in 2010 to 55.0 % (95 % CI 53.9–56.1 %) in 2013, adjusted *p* < 0.001 (using data from all clusters or only clusters participating all 4 years).

### Signs and symptoms used to determine whether the infant had pSBI

**Fig. 4:**
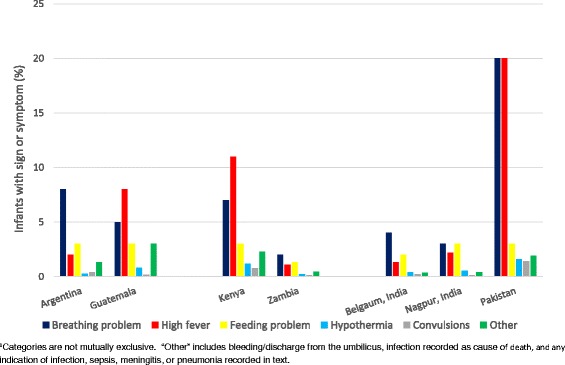
Signs and symptoms of possible severe bacterial infection reported in all live born infants by study site

Across the sites, 60–72 % of neonates/young infants with pSBI met criteria for pSBI based on a single sign or symptom, while the remaining babies met criteria based on multiple symptoms (20–31 % of infants with pSBI had 2 signs; 10 % or less had 3 or more signs, depending on study site). Breathing problems and/or high fever were the most frequently reported symptoms at each study site, followed by feeding problems (Fig. 4). In Pakistan, the percent of all infants reported with breathing problems was more than double that reported in the other sites (20 % versus 2–8 % of all infants; 11 % with breathing problems as the only sign versus 1–4 % at the other sites) and the percent with high fever was higher (20 % versus 1–11 %; 13 % with fever as the only sign versus 1–6 % at the other sites). Combinations involving breathing problems, feeding problems, and/or high fever were most frequent when pSBI was determined based on multiple symptoms. Hypothermia, convulsions, and bleeding/discharge from the umbilicus were each reported for 2 % or less of infants at all sites. An indication of infection, sepsis, meningitis, or pneumonia was reported for <1–2 % of infants depending on study site.

### Case fatality and trends in case fatality rates

**Fig. 5:**
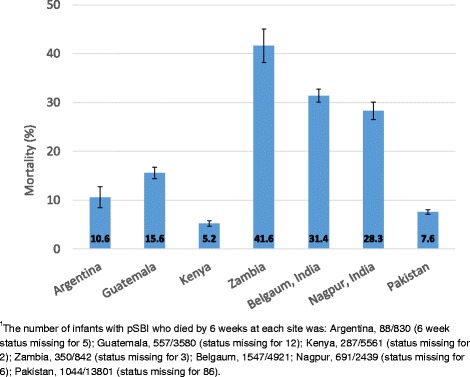
Case fatality rates in neonates with possible severe bacterial infection (pSBI) during the first 6 weeks of life by study site

Of the 32,088 neonates with pSBI during the first 6 weeks, 27,410 (85 %) survived to 6 weeks of age, 4,564 (14 %) died, and 6–week status was missing for 114 (<1 %). In contrast, only 0.5 % of infants without pSBI died by 6 weeks. Case fatality rates in neonates with pSBI varied across sites (Fig. 5). The rate was lowest in Kenya where 5.2 % (95 % CI 4.6–5.8 %) of infants with pSBI had died by 6 weeks of age and highest in Zambia where 41.6 % (95 % CI 38.2–45.0 %) had died. There were no statistically significant changes in the case fatality rates in infants with pSBI in the first 6 weeks of life during the 4 year study period at any site. Case fatality rates associated with signs and symptoms varied by site but generally showed that neither hyperthermia nor umbilical discharge as a single sign is a risk factor for mortality while multiple signs of pSBI is (Table 5).

**Table 5:** Case fatality rates in infants with pSBI in the first 6 weeks of life by pSBI criteria

Variable	Argentina	Guatemala	Kenya	Zambia	Belgaum, India	Nagpur, India	Pakistan
Number of infants with pSBI and non-missing status	830	3,580	5,561	842	4,921	2,439	13,801
Single sign/symptom, n/N (%) died							
Breathing problem/difficulty breathing	26/316 (8.2)	61/561 (10.9)	63/917 (6.9)	59/190 (31.1)	577/2,153 (26.8)	156/584 (26.7)	250/4,395 (5.7)
Feeding problem/stopped suckling/feeding	2/55 (3.6)	31/162 (19.1)	16/139 (11.5)	40/87 (46.0)	102/346 (29.5)	67/319 (21.0)	31/203 (15.3)
High fever	1/88 (1.1)	18/1,431 (1.3)	5/1,814 (0.3)	21/156 (13.5)	7/707 (1.0)	8/592 (1.4)	40/4,852 (0.8)
Hypothermia	0/6 (0.0)	11/85 (12.9)	4/88 (4.5)	4/11 (36.4)	34/124 (27.4)	7/32 (21.9)	26/141 (18.4)
Convulsions	0/3 (0.0)	1/8 (12.5)	2/34 (5.9)	1/10 (10.0)	27/60 (45.0)	7/14 (50.0)	16/89 (18.0)
Bleeding/pus-like discharge from umbilicus	0/42 (0.0)	0/135 (0.0)	1/297 (0.3)	1/39 (2.6)	2/46 (4.3)	0/36 (0.0)	0/252 (0.0)
Infection diagnosis or cause of death	1/14 (7.1)	26/152 (17.1)	13/68 (19.1)	24/25 (96.0)	72/80 (90.0)	23/27 (85.2)	7/9 (77.8)
>1 sign/symptom, n/N (%) died	58/306 (19.0)	409/1,046 (39.1)	183/2,204 (8.3)	200/324 (61.7)	726/1,405 (51.7)	423/835 (50.7)	674/3,860 (17.5)

## Discussion

In a cohort of 248,539 infants born alive between 2010 and 2013 at 7 population based Global Network sites in Latin America, Africa, and Asia 32,088 (13 %) neonates met symptomatic criteria for pSBI. The incidence of pSBI in the first 6 weeks of life, trends in incidence, and in case fatality rates in the first 6 weeks for infants with pSBI varied widely across sites with a 10–fold variation in incidence of pSBI from 3 % (Zambia) to 36 % (Pakistan) and an 8 fold variation in case fatality from 5 % (Kenya) to 42 % (Zambia). Our incidence estimates are in range or somewhat higher than the upper range recently estimated by Seale et al. [7] in a systematic review and meta-analysis of global incidence of pSBI in 2012 (7.6 %, 95 % confidence interval 6.1–9.2 %), except for our two extreme sites, Zambia (lower rates) and Pakistan (higher rates). Unlike Seale et al., we included all live born infants not just those with birth weight ≥ 1,500 g, which may have comparatively inflated our rates because low birth weight babies may be premature and may be more likely to have breathing problems that are not caused by pSBI. Our incidence estimates are likely higher than Seale’s in some cases because we were unable to match the most common sign of pSBI per YICSS, respiratory rate of 60 breaths per minute, and had to use a less specific data point of breathing problems and difficulty breathing. It is likely that these respiratory variables increased the proportion of our pSBI cases that had respiratory distress due to preterm birth or birth asphyxia, both of which are difficult to distinguish from possible severe bacterial infection, particularly in the first few days of life. Our case fatality rates are within the range estimated by Seale et al. (9.8, 95 % confidence interval 7.4–12.2 %) for Argentina and Pakistan, lower for Kenya and higher for Guatemala, Zambia, and the two Indian sites. Although a common protocol was used for prospective data collection, we note that Zambia had the lowest incidence of pSBI but the highest case fatality rate, suggesting that signs and symptoms may have been underreported in this site. In contrast, Pakistan had the highest incidence of pSBI and one of the lower case fatality rates, suggesting there may have been over-reporting of respiratory symptoms or that these symptoms were due to the higher rates of preterm birth and birth asphyxia reported in Pakistan [14]. Overall, our estimates, on average, align with Seale et al. [7], which lends support to the credibility of these findings particularly as these data from the studies contributing to Seale’s paper and the MNH registry (as described in the methods above) were obtained in very different ways. However, the wide variations in pSBI incidence and case fatality rates, even in our prospective registry with trained data collectors, indicate continued need for objective criteria to make a diagnosis of pSBI.

Between 2010 and 2013 there were significant increases in facility deliveries and survival of neonates born preterm in our study sites as well as in the countries our sites represent. Against this background, we found decreases in the incidence of pSBI in the first 6 weeks of life during the period in Argentina, Kenya, and Nagpur, India after accounting for clustering and maternal and neonatal factors. Argentina was the one site where nearly all infants were born in hospital throughout the study period, and the decrease in Kenya occurred among infants born at home as well as in a facility suggesting that changes at these sites cannot be attributed simply to facility birth. In contrast, in Nagpur, India, the decrease in pSBI rate occurred among facility births alone with no significant change in pSBI incidence among infants born at home. While facility births increased from 49 to 62 % of all births in Pakistan, pSBI incidence increased during the period for infants born in a facility as well as for those born at home, after accounting for birth weight and other factors. Reporting or diagnostic changes may have contributed to the increase in Pakistan.

Our prospective study has several important strengths. First, we attempted to define pSBI based on the presence of any one of seven signs found to be predictive of severe illness in young infants [10]. However, we were limited in our ability to match all of these signs, mainly respiratory rate of 60 breaths or more per minute, severe chest indrawing, and movement when stimulated because they were not included in the MNH Registry. We recognize the overlap that breathing problems and difficulty breathing have with problems of prematurity, birth asphyxia, and non-bacterial illnesses. However, the proportions of infants with specific signs and symptoms are highly consistent with the ranges published in recent clinical trials that assessed community based treatment of neonatal sepsis [3,4]. There was extensive training on identification of signs and symptoms of pSBI in these trials, but there was still wide variation in the proportion of infants with signs such as chest indrawing across the study sites. Our data are also limited by lack of microbiologic confirmation in the rural communities and facilities. Further, we did not have the precise details of timing of the symptoms in the first 42 days of life, so it is possible that our incidence estimates are higher than in other studies reporting pSBI between day 0–27 of life and lower than in studies reporting pSBI in young infants between day 0–59 of life. We also do not have information on precise treatment practices by site for access to health care facilities, care seeking, antibiotic access across and over time and how these variables may have impacted case fatality rates. Finally, the MNH registry does not currently collect reliable information on referral practices because the ability to refer infants depends on the availability of referral facilities. Similarly, location of death is not captured currently and would be difficult to interpret because of variable access to referral facilities across the Global Network sites. We recognize that many of our limitations could have resulted in misclassification of pSBI that could have varied by site and over time, but to move forward, improved diagnostics for pSBI are urgently needed. Despite these limitations, the incidence of pSBI at each site is likely indicative of a significant portion of the neonatal illness burden given that infants identified as having pSBI were at greatly increased risk of death during the first 6 weeks of life compared to other infants. We believe that our data are generalizable based on the similarity with the Seale data and the standardized data collection across the 7 Global Network sites.

The high burden of pSBI in neonates and young infants, regardless of definitions and changes in location of birth, is an important reminder that investment in ways to reduce delays in early recognition /diagnosis and treatment of pSBI continues to be needed and is consistent with the research priorities outlined in the Every Newborn Action Plan [5]. Priorities for the future include ways to improve early detection of pSBI in the community, risk stratification, early access to referral of those at the greatest risk of mortality and ways to prevent pSBI particularly in the era of increased delivery of babies in health care facilities.

## Conclusions

In a prospective population based registry with trained data collectors, there were wide variations in the reported incidence and case fatality of pSBI in rural communities and in trends over time. Regardless of these variations, the burden of pSBI is still high and strategies to implement timely diagnosis and treatment are still urgently needed to reduce neonatal mortality.
